# Process quality indicators in family medicine: results of an international comparison

**DOI:** 10.1186/s12875-015-0386-7

**Published:** 2015-12-02

**Authors:** Danica Rotar Pavlič, Maja Sever, Zalika Klemenc-Ketiš, Igor Švab

**Affiliations:** Department of Family Medicine, University of Ljubljana, Medical Faculty, Poljanski nasip 58, 1000 Ljubljana, Slovenia; Statistical Office of the Republic of Slovenia, Litostrojska 54, 1000 Ljubljana, Slovenia; Department of Family Medicine, Medical Faculty, University of Maribor, Taborska 8, 2000 Maribor, Slovenia

**Keywords:** Family physician, Process quality, Country, Comparison

## Abstract

**Background:**

The aim of our study was to describe variability in process quality in family medicine among 31 European countries plus Australia, New Zealand, and Canada. The quality of family medicine was measured in terms of continuity, coordination, community orientation, and comprehensiveness of care.

**Methods:**

The QUALICOPC study (Quality and Costs of Primary Care in Europe) was carried out among family physicians in 31 European countries (the EU 27 except for France, plus Macedonia, Iceland, Norway, Switzerland, and Turkey) and three non-European countries (Australia, Canada, and New Zealand). We used random sampling when national registers of practitioners were available. Regional registers or lists of facilities were used for some countries.

A standardized questionnaire was distributed to the physicians, resulting in a sample of 6734 participants. Data collection took place between October 2011 and December 2013. Based on completed questionnaires, a three-dimensional framework was established to measure continuity, coordination, community orientation, and comprehensiveness of care. Multilevel linear regression analysis was performed to evaluate the variation of quality attributable to the family physician level and the country level.

**Results:**

None of the 34 countries in this study consistently scored the best or worst in all categories. Continuity of care was perceived by family physicians as the most important dimension of quality. Some components of comprehensiveness of care, including medical technical procedures, preventive care and health care promotion, varied substantially between countries. Coordination of care was identified as the weakest part of quality. We found that physician-level characteristics contributed to the majority of variation.

**Conclusions:**

A comparison of process quality indicators in family medicine revealed similarities and differences within and between countries. The researchers found that the major proportion of variation can be explained by physicians’ characteristics.

**Electronic supplementary material:**

The online version of this article (doi:10.1186/s12875-015-0386-7) contains supplementary material, which is available to authorized users.

## Background

Primary healthcare (PHC) is the foundation of healthcare systems in many parts of the world [1, 2]. Family physicians (FPs) are crucial stakeholders in delivering national health policy through primary care [3–6].

Although the World Health Organization (WHO) definition of primary healthcare is widely accepted, there are differences between countries [2]. A recent WHO World Health Report emphasized the importance of measuring health system performance [7]. Variation in performance occurs in the structure, processes, and outcomes of care; causes of variation include systemic factors, reimbursement, service organization and capacity, cultural factors, and disease epidemiology [8, 9]. Studies at the international level [10, 11] revealed striking differences between practice systems in terms of incentives, practice information capacity, access, the use of teams in quality policy, detection of quality and safety problems, staff and patient safety, inclusion of patients’ perspectives, and the length of consultations [12].

Much is known about specific parameters, such as specific disease management [13–15], guidelines, and outcomes of treatment [16], and researchers have insight into the workload [17, 18], but there is a lack of complex multilevel comparisons between countries. Analyses usually focus on secondary and tertiary care [19, 20]. An important development is also extending the scope of reporting particularly into primary care [21].

The broad concept of quality in healthcare [22, 23] can be divided into the features of structure, process, and outcome. Four dimensions are specially addressed: continuity, coordination, comprehensiveness of care, and community orientation [23–26]. Continuity of care includes informational, relational, and management continuity. Coordination reflects the organization of services between different levels of care. Comprehensiveness is defined by the scope of practice and a wide range of services provided by FPs [27]. Community orientation is important for a feasible and sustainable health system [28].

This study describes the variability in process quality in family medicine among 31 European countries plus Australia, New Zealand, and Canada.

## Methods

### Study design

The Quality and Costs of Primary Care in Europe study (QUALICOPC) was a descriptive cross-sectional study designed to collect information about the practice setting, the services provided, patient values, and patient experience. This study presents the results of participating FPs. The detailed rationale, design, and methods of QUALICOPC have been previously described [29, 30].

### Setting

The study was held among FPs in 31 European countries (the EU 27 except for France, plus Macedonia, Iceland, Norway, Switzerland, and Turkey) and three non-European countries (Australia, Canada, and New Zealand). All 10 provinces in Canada participated. In the United Kingdom, the study took place only in England. At the beginning of the project, we also included France. Due to various setbacks, we did not succeed in collecting data and therefore France was excluded from the study.

### Participants

At the coordinators’ meeting, we decided that the number of FPs sampled should be large enough to obtain a response from at least 220 FPs in each country (one FP per practice). Thus, the size of the sample depended on the expected participation of FPs. For example, if the national coordinator expected 25 % of the FPs contacted to participate, the original sample size had to include at least 880 FPs. In countries with a very small population, the desired number of FPs surveyed was smaller (80 to 100). FPs were invited to participate using various methods: e-mail, letters, telephone calls, personal contacts, and advertisements.

We aimed for a nationally representative sample of FPs. If national registers of practitioners were available, we used random sampling to select practitioners. In countries with only regional registers, random samples were drawn from regions that represented the national setting. If no registers existed, but only lists of facilities in a country, a random selection was made from such lists [29, 31, 32]. The sampling and recruitment procedures with response rates per country are presented in Appendix A: Table 6.

### Questionnaire

The original English version of the questionnaire was translated into the various national languages. A professional translator created an independent back-translation (the final questionnaire is available in Additional file 1). FPs answered questions related to the primary care process (i.e., continuity, coordination, community orientation, and comprehensiveness of care). Ethical approvals were acquired in line with the legal requirements in each country. Dimensions (Table 1) were composed from indicators based on a literature review [6, 30]. This review also contributed to different scales of a specific indicator. Higher values on the score indicated a higher level of process quality.

**Table 1:** Selected indicators and dimensions of process quality in primary healthcare

Dimension	Definition of indicator	Scale
Continuity of care	Medical recordkeeping: inclusion of important health information	0 to 1
	Medical recordkeeping: regularity of keeping medical files	0 to 1
	Informational continuity of care with primary care: receiving records from previous doctor	1 to 3
	Informational continuity of care with secondary care: receiving discharge report	1 to 5
Coordination of care	Skill mix: disciplines in practice	0 to 1
	Integration of primary and secondary care: asking other specialists for advice	1 to 3
	Collaboration with other providers	1 to 3
Community orientation	Reporting potential repeated accidents in an industry, frequent respiratory problems in patients living near a particular industry, and repeated cases of food poisoning among people living in a certain district to an authority	1 to 4
Comprehensiveness of care	Medical equipment available	0 to 1
	First contact for common health problems	1 to 4
	Treatment and follow-up diseases	1 to 4
	Medical technical procedures and preventive care	1 to 4
	Healthcare promotion	0 to 1

### Data collection and analysis

Data collection took place between October 2011 and December 2013. The FP questionnaire was completed by 6734 FPs. Statistical analyses were performed using IBM SPSS Statistics for Windows (Version 21.0, Released 2012. Armonk, NY: IBM Corp). The confidence level was set at *p* < 0.05. All indicators were rescaled to a common scale (a *z*-score) with an average of zero and a standard deviation of one.

Standardized scores were used for inter-indicator comparisons and calculation of the composites for each dimension. The composite score of a dimension was defined as the mean of *z*-scores for relevant indicators. The Pearson correlation of each dimension in relation to the corresponding composite was also examined. We computed the means and standard deviations of all indicators at the country level and for the entire sample to determine discriminative power. To gain more insight into country patterns, the 34 countries were plotted on a graph using the composite scores for continuity, coordination, community orientation, and comprehensiveness. We used linear mixed models to decompose the variance of the composites into two components: the FP level and the country level. We estimated null and random intercept models by using variance component as the covariance structure and maximum likelihood. The indicators of process quality were defined as dependent variables. To assess the proportion of variance by country, we computed the interclass correlations (ICC).

### Ethics approval

Ethics approval was acquired in accordance with the legal requirements in each country. We listed the full name of every ethics committee which approved the study protocol in Additional file 2. There are 6 countries where there was no requirement to go through the ethics procedure. The explanation for those countries is given in the same additional file, for example the national coordinator of Slovakia consulted with the Council of the Slovak Society of General Practice. It was confirmed that there is no statutory requirement for ethical approval for this study.

Depending on the national requirements, written or oral informed consent was requested. The general procedure was that FPs were invited via letter, e-mail or telephone and gave their consent to participate in the study. Patients were invited by the fieldworker or practice staff to complete a questionnaire. All participants were informed about the study and participation was voluntary.

## Results

### Demographic characteristics

We received completed questionnaires from 6734 FPs. The average age of an FP was 50.3 ± 9.7 years. FPs were predominantly female in 15 countries (91.6 % in Estonia, followed by Lithuania, Latvia, Romania, Macedonia, and Slovenia). The least female workforces were observed in Switzerland, the Netherlands, Iceland, Austria, Malta, and Ireland. Greece had the youngest FPs (average 43.3 years), and Italy the oldest (57.0 years). A total of 2534 FPs (38.0 %) were 55 or older, ranging from 5.4 % (*n* = 15) in Turkey to 73.4 % (*n* = 149) in Italy. The largest proportion of FPs continuing to work after age 65 was in Hungary (14.9 %) and Slovakia (11.3 %).

### Continuity and coordination of care by country

**Fig. 1:**
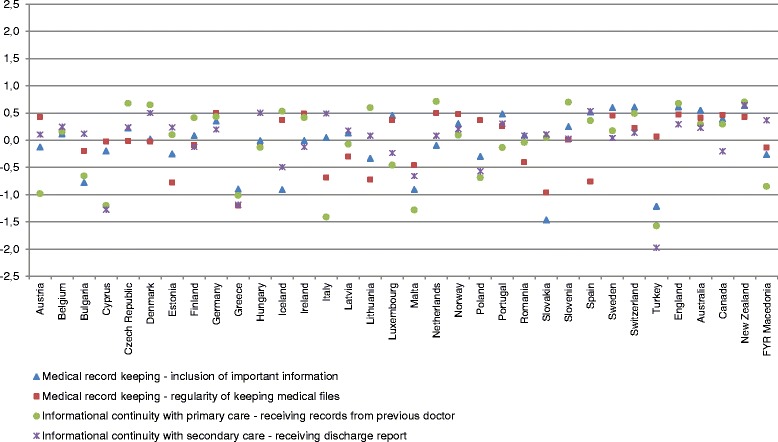
Standardized performance of continuity of care indicators by country. Notes: In Figs. 1 and 2, indicators were rescaled to a common scale with an average of zero and standard deviation of one (i.e., *z*-scores). If a *z*-score was positive, its corresponding raw score was above the mean. If a *z*-score was negative, its corresponding raw score was below the mean

Belgium, Germany, Norway, Slovenia, Sweden, Switzerland, England, Australia, and New Zealand stand out as excellent for continuity of care, with positive scores for all four indicators. Cyprus, Greece, and Malta had negative *z*-scores for the same four indicators (Fig. 1).

**Fig. 2:**
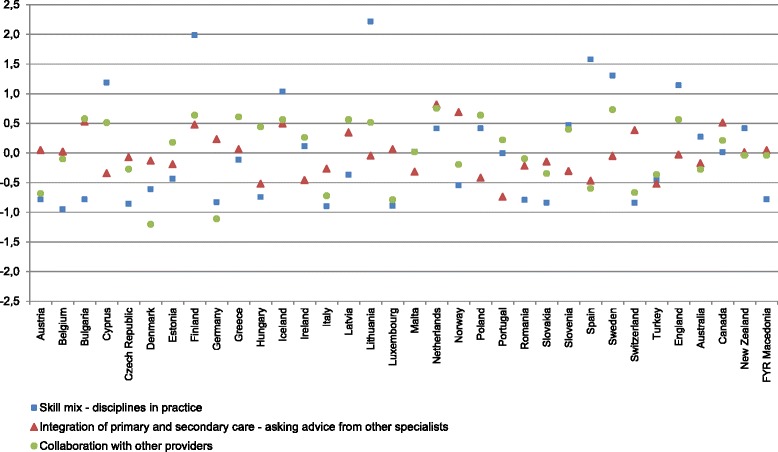
Standardized performance on coordination of care indicators by country

Variation of coordination of care indicators by country are presented in Fig. 2. Skill mix, integration, and collaboration differ from country to country. On the other hand, countries are grouped differently here than in Fig. 1. The highest scores for collaboration with other providers were reported in Sweden, the Netherlands, Finland, Poland, and Greece.

### Variation of dimensions between countries

The dimensions were calculated as composites and are composed of individual indicators; they show the general state of continuity, coordination, community orientation, and comprehensiveness of care in a specific country. Composite scores make it possible to assess which dimension is the most variable in the group of 34 countries (Table 2). Community orientation, with an absolute range of 1.825, was the most variable dimension, followed by continuity of care (1.798), coordination (1.683), and comprehensiveness of care (1.565).

**Table 2:** Composite scores of process quality dimensions by country by mean and standard deviation

Country	Continuity of care	Coordination of care	Community orientation	Comprehensiveness of care
Europe				
Austria	−0.149 (0.465)	−0.468 (0.524)	0.066 (0.905)	0.126 (0.589)
Belgium	0.170 (0.427)	−0.339 (0.563)	−0.017 (0.888)	0.001 (0.402)
Bulgaria	−0.401 (0.605)	0.106 (0.637)	0.050 (0.927)	−0.237 (0.492)
Cyprus	−0.678 (0.669)	0.441 (0.506)	−1.284 (1.685)	−0.827 (0.464)
Czech Republic	0.282 (0.321)	−0.398 (0.453)	−0.050 (0.813)	−0.766 (0.404)
Denmark	0.288 (0.377)	−0.649 (0.387)	−0.058 (0.840)	0.423 (0.311)
England	0.513 (0.225)	0.561 (0.475)	0.204 (0.854)	0.692 (0.409)
Estonia	−0.158 (0.451)	−0.149 (0.480)	−1.159 (1.293)	−0.184 (0.342)
Finland	0.075 (0.433)	1.034 (0.511)	0.051 (0.706)	0.372 (0.522)
Germany	0.371 (0.316)	−0.574 (0.468)	−0.364 (0.917)	0.263 (0.391)
Greece	−1.085 (0.905)	0.194 (0.542)	0.325 (1.011)	0.045 (0.590)
Hungary	0.066 (0.467)	−0.276 (0.437)	−0.728 (1.635)	−0.440 (0.462)
Iceland	−0.139 (0.373)	0.702 (0.532)	0.191 (0.734)	0.093 (0.468)
Ireland	0.197 (0.328)	−0.026 (0.453)	−0.055 (0.855)	0.476 (0.424)
Italy	−0.394 (0.534)	−0.617 (0.546)	0.304 (0.819)	−0.601 (0.359)
Latvia	−0.020 (0.516)	0.175 (0.509)	−0.326 (1.105)	−0.410 (0.413)
Lithuania	−0.073 (0.436)	0.896 (0.650)	0.049 (0.987)	0.021 (0.476)
Luxembourg	0.038 (0.362)	−0.537 (0.597)	−0.023 (0.966)	−0.303 (0.471)
Macedonia	−0.221 (0.516)	−0.259 (0.516)	0.083 (0.960)	−0.362 (0.428)
Malta	−0.840 (0.588)	−0.101 (0.547)	0.085 (0.935)	−0.410 (0.442)
Netherlands	0.305 (0.264)	0.657 (0.502)	0.247 (0.843)	0.574 (0.393)
Norway	0.273 (0.318)	−0.019 (0.475)	0.542 (0.814)	0.627 (0.300)
Poland	−0.306 (0.509)	0.211 (0.648)	0.053 (1.115)	−0.546 (0.396)
Portugal	0.227 (0.433)	−0.175 (0.392)	−0.306 (0.978)	−0.216 (0.386)
Romania	−0.065 (0.502)	−0.368 (0.548)	0.024 (1.285)	−0.532 (0.489)
Spain	0.189 (0.456)	0.170 (0.544)	0.236 (0.761)	0.369 (0.403)
Slovakia	−0.576 (0.781)	−0.468 (0.485)	0.093 (1.007)	−0.770 (0.528)
Slovenia	0.248 (0.323)	0.187 (0.484)	0.087 (0.847)	0.294 (0.431)
Sweden	0.317 (0.319)	0.665 (0.473)	−0.258 (0.993)	0.738 (0.296)
Switzerland	0.368 (0.333)	−0.367 (0.459)	−0.039 (0.843)	0.490 (0.390)
Turkey	−1.194 (0.617)	−0.447 (0.494)	0.477 (0.868)	−0.748 (0.444)
Non-European				
Australia	0.363 (0.344)	−0.0620 (0.527)	−0.146 (0.910)	0.351 (0.418)
Canada	0.239 (0.327)	0.25 (0.659)	−0.104 (0.852)	0.131 (0.465)
New Zealand	0.604 (0.199)	0.129 (0.480)	0.122 (0.736)	0.736 (0.372)

*Notes*: Composite scores of dimensions are calculated as the mean values of standardized indicators. A positive score indicates that the average value is above the mean, and a negative score indicates below the mean. The minimum and maximum values in Table 2 are the lowest and highest scores recorded for each dimension. The range is the measure of the absolute difference between the minimum and maximum values

### Clusters of countries

Countries were combined into clusters according to their rankings based on composite scores. Table 3 shows which countries were grouped into the top and bottom five positions. The ranks of Australia, Canada, and New Zealand are also shown separately because they are non-European countries. The country with best “top five” positions was the Netherlands, and Cyprus and Slovakia shared the worst place among “bottom five” countries.

**Table 3:** Range of composite scores with country rankings by process quality dimensions

	Continuity of care	Coordination of care	Community orientation	Comprehensiveness of care
Minimum	−1.194	−0.649	−1.284	−0.827
Maximum	0.604	1.034	0.542	0.738
|Range|	1.798	1.683	1.825	1.565
Top five countries	New Zealand	Finland	Norway	Sweden
	England	Lithuania	Turkey	New Zealand
	Germany	Iceland	Greece	England
	Switzerland	Sweden	Italy	Norway
	Australia	Netherlands	Netherlands	Netherlands
Bottom five countries	Turkey	Denmark	Cyprus	Cyprus
	Greece	Italy	Estonia	Slovakia
	Malta	Germany	Hungary	Czech Republic
	Cyprus	Luxembourg	Germany	Turkey
	Slovakia	Slovakia	Latvia	Italy
Non-European				
Australia	5th	18th	27th	11th
Canada	12th	8th	26th	14th
New Zealand	1st	14th	10th	2nd

### Contribution of FP characteristics and country characteristics to total variability in quality

In addition to country rankings, this study sought to estimate how much variability in process quality indicators stems from within-group differences (i.e., individual level) how much from between-group differences (i.e., country level; Table 4).

**Table 4:** Multilevel model, which includes variance in individual FP level and country-system level

Dimension	Indicator	Constant	FP-level variance	Country-level variance	ICC (%)
Continuity of care	Inclusion of important health information in medical records	0.887	0.016	0.007	28.8
	Regularity of keeping medical record files	0.773	0.135	0.038	22.2
	Receiving records from previous primary care doctor	2.430	0.308	0.250	44.8
	Receiving discharge reports from secondary care	3.767	1.012	0.434	30.0
Coordination of care	Skill mix: disciplines in practice	0.223	0.006	0.036	84.9
	Asking other specialists for advice	1.688	0.185	0.029	13.7
	Collaboration with other providers	1.917	0.060	0.025	29.3
Community orientation	Reporting potential public health threats in a particular district to an authority	3.255	0.295	0.043	12.7
Comprehensiveness of care	Medical equipment available	0.534	0.018	0.040	68.3
	First contact for common health problems	2.904	0.175	0.087	33.2
	Treatment and follow-up of diseases	3.268	0.169	0.077	31.3
	Medical technical procedures and preventive care	2.114	0.199	0.452	69.4
	Healthcare promotion	0.193	0.019	0.009	30.8

*Notes*: Multilevel models were estimated using raw data. Tests of significance for all models showed that the average (constant) statistically differed from zero (*p* = 0.000 < 0.05). ICC values were calculated as percentage of country level variance in total observed variance

The main share of the total variation is explained by individual FP characteristics. The ICC values in 10 out of 13 indicators show that the majority of total variability is based on the variability between the FPs.

In addition, the overall contribution of FP characteristics to variation of quality related to the four dimensions was estimated (Table 5). The ICC values revealed that the main part of the total variation is explained by country characteristics only in the case of the comprehensiveness of care dimension. The other three dimensions (i.e. continuity, coordination, community orientation) show that majority of total variability stems from the variability between FPs. The smallest overall contribution of country-system features to variation in quality was assessed for the community orientation dimension.

**Table 5:** Multilevel model based on composite scores for quality dimensions

Dimension	Constant	FP-level variance	Country-level variance	ICC (%)
Continuity of care	−0.034	0.216	0.185	46.2
Coordination of care	0.002	0.279	0.199	41.7
Community orientation	−0.045	0.906	0.132	12.7
Comprehensiveness of care	−0.015	0.190	0.227	54.5

*Notes*: ICC values were calculated as the percentage of country-level variance in total observed variance

## Discussion

The advantage of the study lies in the fact that it is a comparison between 34 countries, which is a much larger number compared to other international studies [33, 34], which usually do not have an accurate inventory selection of physicians in individual countries and their response rates [33–35].

A previous study of this group assessed dimensions of PC and indicators for these dimensions [19]. This study sought to comprehensively evaluate primary care systems by perceptions of FPs (FP questionnaire). Compared to QUALICOPC, PHAMEU (Primary Health Care Activity Monitor for Europe) was more oriented toward the system and structure of primary care, and this is why we cannot draw direct parallels between the two studies. The PHAMEU countries with the strongest primary care structure were Denmark, Finland, Italy, the Netherlands, Portugal, Romania, Slovenia, Spain, and the United Kingdom. Countries with a relatively weak primary care structure in all three dimensions were Bulgaria, Cyprus, the Czech Republic, Greece, Iceland, Luxembourg, Poland, and Slovakia [36].

### Continuity

Continuity of care is strongly related to the organization of primary care, which should not be fragmented and should have few entry points into the overall healthcare system [21].

Composite scores of continuity of care were ranked as unfavourable in Turkey and Greece, Malta, Cyprus, and Slovakia. The best continuity composite scores were calculated in New Zealand and England. This diversity might be a consequence of different conditions of primary care delivery. Our results confirm the results of Kringos et al., who already pointed out problems of continuity in Turkish general practice, suggesting reductions in list size [37, 38] and discussions about the future of FPs in Greece, which have resulted in proposals to increase quality and efficiency [39].

### Coordination

Well-coordinated care is important for patients with chronic illnesses, especially those with multiple conditions. Patients with chronic illnesses do better in countries with strong primary care infrastructures, even if deficiencies exist in all countries [40], especially in the transition after hospital discharge, with inadequate coordination between various physicians and in weak efforts engaging or supporting patients to manage their own health.

In our study, Dutch, Finish, Lithuanian, Icelandic, and Swedish FPs reported positive experiences with coordination of care. Coordination in Germany, Denmark, Italy, Luxembourg, and Slovakia was not assessed as good. Schoen et al. [26] found a different situation. Physicians in Germany did not believe that their patients experience coordination problems. German doctors reported the lowest rates of concern on three out of four coordination questions. The differences between the results published by Schoen and our results can be explained by different measurement instruments. Whereas Schoen’s assessment of coordination primarily applied to the timely transfer of diagnoses between the FP and other specialists, our survey also included questions about integration between various levels and the frequency of direct consultations between FPs and specialists.

Small private practices in Denmark may impede coordination of care and lead to a “culture of individualism.” Recent Danish initiatives are trying to improve coordination between the primary and secondary healthcare sectors [41].

### Community orientation

Community orientation is considered one of the key features of good PHC [42–44].

Nevertheless, this showed the most variation between countries. This probably reflects the differences in health systems in the countries studied and the historical background against which the systems were shaped in the past.

### Comprehensiveness

In this study, FPs from Sweden, New Zealand, England, Norway, and the Netherlands evaluated comprehensiveness of care as very good. The opposite situation was found in Cyprus, Slovakia, the Czech Republic, Turkey, and Italy. Schäfer et al. [32] found a high need for improvement in comprehensiveness in Cyprus and Malta, and a medium need in Turkey.

### Overall

Overall, none of the 34 countries in this study consistently scored the best or worst in all categories of quality measured. The Netherlands was the only country that achieved the best average ranks in all four dimensions. New Zealand placed in the “top five” position for continuity and comprehensiveness of care, but was not as good for coordination of care and community orientation. Cyprus and Slovakia placed in the “bottom five” position three times. Another study found that “within a given health care system, doctors’ personal and practice characteristics explained only a small part of the variance in attitudes toward the provision of personal continuity of care” [45]. We found that that more than half of the variance was explained by physicians’ characteristics and not the characteristics of the country healthcare system. The results seem reasonable because the FPs in our study primarily evaluated their own practices and not general national health services. Future intervention to improve the quality of primary care should focus on the FP-level characteristics identified in this study.

### Strengths and limitations

We believe that the 6734 participating FPs, selection criteria, and response rates are important achievements of this study. The respondents were considered representative of the national FP population. However, the target of 220 FPs was not reached in some smaller countries (e.g., Cyprus, Iceland, Luxembourg, and Malta). In a large country like Canada, GPs were sampled from a nationally representative region [31]. We sought to acquire a qualitatively and quantitatively sufficient response in each country. The variation in response rates between countries was partly due to structural challenges. In fact, in many participating countries there is not a national central register of FPs and this is why we had to approach the FPs individually [31]. In any case, we tried to maximize the responsiveness of FPs and we considered recommendations to increase the response rates [46].

The limitations of this study lie in self-reporting of FPs. We do not know whether the FPs in all countries were equally critical of their primary care situation. It is possible that physicians in some countries that have high-quality primary healthcare are more critical of their own work than physicians in countries with less optimal quality of care. Important information about the quality of primary healthcare can also be obtained from patients’ assessments of their own care. An analysis based on this data will be presented in subsequent studies and articles.

There were also limitations in the data set. The specific set of variables used in this analysis was selected based on the initial reduction of all variables by NIVEL (the Netherlands Institute for Health Services Research). Additional issues in the FP questionnaire could have been included to broaden the dimension of community orientation. On the other hand, doctors that inform the community about possible health threats usually also coordinate processes that protect the community.

Another limitation may have been in the way composite scores were weighted. Composites summarize data from many quality indicators to aid in understanding complex processes [47]. We are aware that weighting systems are arbitrary. On the other hand, composite measures are increasingly used to assess the universal quality of healthcare.

## Conclusions

There is broad variation in the perception of process quality in family practice. The greatest variation is in community orientation. The main factor in variability seems to be physicians’ characteristics as defined in this study. This is useful in formulating recommendations for health policy because it has implications for doctors’ training and other policy measures.

## Appendix A

**Table 6:** Sampling and recruitment procedures with response rates per country

Country	Sampling procedure^a^	Recruitment methods	FPs invited	FPs participated	Response rate
Europe					
Austria	B	E-mail, personal contact	3050	173	6%
Belgium	B	Letter, telephone, e-mail	5000	382	8%
Bulgaria	B	Telephone, face-to-face	350	209	60%
Cyprus	A	Letter, telephone	90	67	74%
Czech Republic	B	Letter, telephone, personal contact	520	205	39%
Denmark	B	E-mail	2000	199	10%
England	C	Letter, e-mail	1508	160	11%
Estonia	A	Letter, telephone, e-mail	802	121	15%
Finland	D	Letter, e-mail, telephone, personal contact	1000	270	27%
Germany	B	Letter	3825	223	6%
Greece	D	Telephone, letter	300	206	69%
Hungary	B	E-mail and personal contact	400	209	52%
Iceland	A	Letter and personal contact	95	75	79%
Ireland	D	Letter, e-mail, personal contact, advertisement	2515	158	6%
Italy	E	Telephone	Not known	204	Not known
Latvia	B	Telephone, e-mail	545	205	38%
Lithuania	B	Personal contact, telephone	508	211	42%
Luxembourg	A	Telephone	120	73	61%
Macedonia	B	Letter, e-mail	240	134	56%
Malta	B	Telephone	78	65	83%
Netherlands	B	Letter, e-mail, telephone	1400	224	16%
Norway	E	Letters, telephone, conferences	500	185	37%
Poland	C	Letter, telephone, e-mail	665	206	31%
Portugal	B	Letter, e-mail, telephone	800	203	25%
Romania	B	Letter, telephone, e-mail, personal contact	399	206	52%
Spain	C	E-mail, telephone	500	402	80%
Slovakia	B	Letter, telephone, personal contact	1000	206	21%
Slovenia	B	Letter, telephone, e-mail	1173	194	17%
Sweden	B	Letter	1000	91	9%
Switzerland	B	Letter, telephone	2027	186	9%
Turkey	C	Letter and personal contact	1300	281	22%
Non-European					
Australia	D	Letter	3201	142	4%
Canada	B	Letter, e-mail, telephone	23,671	502	2%
New Zealand	B	Letter	1371	157	11%

*Notes*: ^a^Sampling procedures codes: A) almost entire FP population; B) random national sample (stratified or not); C) random sample in pre-selected regions; D) mixed procedure (random procedure plus selected FPs); E) opportunity sampling and volunteers

## Additional files

Additional file 1:
**Questionnaire for FPs.** (DOC 120 kb) Additional file 2: (DOCX 19 kb)

## Abbreviations

FP: family physician
ICC: interclass correlation
NIVEL: Netherlands Institute for Health Services Research
PHAMEU: Primary Health Care Activity Monitor for Europe
PHC: primary healthcare
QUALICOPC: Quality and Costs in Primary Care
WHO: World Health Organization
