# Supplementary material for: Process quality indicators in family medicine: results of an international comparison
Source: BMC Fam Pract. 2015 Dec 2;16:172. doi: 10.1186/s12875-015-0386-7 (PMC4667500; doi:10.1186/s12875-015-0386-7)
Supplement: Additional file 1: — Questionnaire for FPs. (DOC 120 kb) [file 12875_2015_386_MOESM1_ESM.doc]

Questionnaire for FPs

|  | 1. Are you male or female? | | ❒ Male  ❒ Female | | | |  |
| --- | --- | --- | --- | --- | --- | --- | --- |
|  | 2. What is your year of birth? Please fill in: | | Year of birth: 19__ | | | |  |
|  | 3. Were you born outside this country? | | ❒ No  ❒ Yes | | | |  |
|  | 4. How would you characterise the place where you are currently practising? | | ❒ (Inner) city  ❒ Suburbs  ❒ (Smaller) town  ❒ Mixed urban-rural  ❒ Rural | | | |  |
|  | 5. What is the (estimated) size of your practice population? (In a joint practice: estimate your share of the population).  If you do not have a formal list, please estimate the number of people that normally rely on you for primary medical care. | | Number of patients: ____ | | | |  |
|  | 6. To what extent do you think your practice population compares to the average national level with respect to the following categories:  1. Elderly people (over 70 years)  2. Socially deprived people  3. Immigrants | | Below Average Above Don’t  average average know  ❒ ❒ ❒ ❒  ❒ ❒ ❒ ❒  ❒ ❒ ❒ ❒ | | | |  |
|  | 7. To what extent do you think that the patient turnover in your practice compares to other practices? | | Below Average Above Don’t  average average know  ❒ ❒ ❒ ❒ | | | |  |
|  | 8. How many hours per week do you work as a GP (excluding additional jobs and on-call or out-of-hours services)? | | ___ hours per week | | | |  |
|  | 9. How many of these hours do you spend on direct patient care (consultations, home visits, telephone consultations)? | | ___ hours per week | | | |  |
|  | 10. How many patient contacts do you have on a normal working day?  1. Face-to-face in your office (number)  2. By telephone  3. By e-mail | | ___ per day  ___ per day  ___ per day | | | |  |
|  | 11. How long does a regular patient consultation in your office usually take? | | ___ minutes (Open) | | | |  |
|  | 12. In a normal working week, how many patients do you see:  1. At home visits  2. In hospital  3. In homes for the elderly | | ___ per week  ___ per week  ___ per week | | | |  |
|  | 13. In the past 3 working months (excluding holidays etc.), how often and for how long did you have on call duties during evenings, nights and weekends:  1. During evening(s)  2. During night(s)  3. During weekend days | | ___ times; in total ___hours  ___ times; in total ___hours  ___ times; in total ___hours | | | |  |
|  | 14. Beside your work as a GP in this practice, do you have any other paid professional activities? (multiple answers possible) | | ❒ No  ❒ Yes, as a physician (not GP) for privately paying patients  ❒ Yes, in a residential setting (e.g. nursing home, prison)  ❒ Yes, as a company doctor  ❒ Yes, in teaching/ medical education  ❒ Yes, other | | | |  |
|  | 15. As a GP, are you self-employed or in salaried employment? | | ❒ Salaried employment with centre or authority  ❒ Salaried employment with other GP  ❒ Self-employed with contract(s) with health service or insurance  ❒ Self-employed without contract | | | |  |
|  | 16. For each of the following components please estimate whether they contribute to your income as a GP, and if so, up to what percentage? | | ❒ Salary __%  ❒ Capitation payments (a fixed sum per patient for a certain period of time) __%  ❒ Fee for services from third party payer __%  ❒ Out of pocket payments from patients __%  ❒ Performance payments (for instance related to targets)__ %  ❒ Other sources __% | | | |  |
|  | 17. Can you receive an extra financial incentive or bonus for:  1. Management of patients with diabetes  2. Management of patients with hypertension  3. Achievement of targets for screening or prevention  4. Referral rates below a certain level  5. Having disadvantaged patients in your practice  6. Working in a remote area | | Yes No Don’t know  ❒ ❒ ❒  ❒ ❒ ❒  ❒ ❒ ❒  ❒ ❒ ❒  ❒ ❒ ❒  ❒ ❒ ❒ | | | | |
|  | 18. Do you work alone or in shared accommodation with one or more GPs and/or medical specialists? Please also fill in their number of Full Time Equivalents (FTEs). (For instance: one doctor working 5 days a week and 1 other doctor working 2.5 days a week makes 1.5 FTEs). | | ❒ Alone  ❒ With __ other GPs in shared accommodation  ❒ With __ medical specialist(s) in shared accommodation | | | **FTE**  counting for ___FTE counting for ___FTE | |
|  | 19. Which of the following disciplines are working in your practice/centre?  1. Receptionist/med. secretary  2. Practice nurse  3. Nurse practitioner (function between physician and nurse)  4. Assistant for laboratory work  5. Manager of the centre or practice (not a physician)  6. Community / home care nurse  7. Psychiatric nurse  8. Midwife  9. Physiotherapist  10. Dentist  11. Pharmacist  12. Social worker | | Yes  ❒  ❒  ❒  ❒  ❒  ❒  ❒  ❒  ❒  ❒  ❒  ❒ | | | | |
|  | 20. Do you use clinical guidelines for the treatment of the following?  1.Chronic heart failure  2. Asthma  3. COPD  4. Diabetes | | | | Yes No Not available  ❒ ❒ ❒  ❒ ❒ ❒  ❒ ❒ ❒  ❒ ❒ ❒ | | |
|  | 21. In the past 12 months, have you been involved in a disease management programme for patient with the following chronic conditions? (such programmes are multidisciplinary approaches across practices, often based on protocols).  1.Chronic heart failure  2. Asthma  3. COPD  4. Diabetes | | | | Yes No  ❒ ❒  ❒ ❒  ❒ ❒  ❒ ❒ | | |
|  | 22. In the past 12 months, has the following occurred in your practice/centre?  1. Feedback on your prescriptions or referrals by health authority or insurer?  2. Feedback from colleague GPs (peer review or practice visitation)?  3. Investigation into the satisfaction of your patients? | | | Yes No  ❒ ❒  ❒ ❒  ❒ ❒ | | | |
|  | 23. Who usually decides about where the patient is referred to? | | | ❒ I do  ❒ The patient does  ❒ It is a shared decision | | | |
|  | 24. To what extent do you take into account the following considerations:  1. I leave it to the patient to decide whom to go to  2. The travel distance for the patient  3. Previous experiences with the medical specialist  4. Comparative performance information on medical specialists  5. Waiting time for the patient  6. Costs for the patient | | | Always Sometimes Never  ❒ ❒ ❒  ❒ ❒ ❒  ❒ ❒ ❒  ❒ ❒ ❒  ❒ ❒ ❒  ❒ ❒ ❒ | | | |
|  | 25. Please tick the equipment used in your practice by yourself or your staff: | | Laboratory  ❒ Hemoglobinometer  ❒ Any blood glucose test set  ❒ Any cholesterol meter  ❒ Blood cell counter  Imaging  ❒ Ophthalmoscope  ❒ Proctoscope  ❒ Otoscope  ❒ Gastroscope  ❒ Sigmoidoscope  ❒ X‐ray  ❒ Ultrasound for abdomen/ fetus  ❒ Microscope  Functions  ❒ Audiometer  ❒ Bicycle ergometer  ❒ Eye tonometer  ❒ Peak flow/ PEF meter  ❒ Spirometer  ❒ Electrocardiograph  ❒ Blood pressure meter  ❒ Infusion set  ❒ Doctor’s bag for emergencies and home visits  Other  ❒ Urine catheter  ❒ Coagulometer  ❒ Set for minor surgery  ❒ Suture set  ❒ Defibrillator  ❒ Disposable syringes  ❒ Disposable gloves  ❒ Refrigerator for medicines  ❒ Resuscitation equipment | | | | |
|  | 26. How do you have access to laboratory facilities? | | ❒ Within my practice/centre  ❒ Easy access outside my practice/centre  ❒ Insufficient access | | | | |
|  | 27. How do you have access to X-ray facilities? | | ❒ Within my practice/centre  ❒ Easy access outside my practice/centre  ❒ Insufficient access | | | | |
|  | 28. What is the distance by road from your (main) practice building to:  1. The nearest GP practice (not in your group or centre)  2. The nearest consultant/outpatient clinic (independent or part of hospital)  3. The nearest general or university hospital | | In the same/Less than/11-20/More than  building 10 kms kms 20 kms  ❒ ❒ ❒ ❒  ❒ ❒ ❒ ❒  ❒ ❒ ❒ ❒ | | | | |
|  | 29. How many hours on an average working day is your practice/centre open for patient care (lunch breaks excluded)? | | ____ hours per working day | | | | |
|  | 30. Is it possible for your patients to visit your practice/centre:  1. After 18h00 (at least once per week)  2. On a weekend day (at least once per month) | | ❒ Yes ❒ No  ❒ Yes ❒ No | | | | |
|  | 31. Outside your practice opening hours, how do your patients have access to (non-emergency) medical services? | | ❒ Not applicable (I am always available for my patients)  ❒ I am available on rota basis with a group of GPs  ❒ I am not available, but other GPs are available on a rota basis  ❒ Other physicians (not GPs) provide out-of hours care  ❒ Other arrangements | | | | |
|  | 32. What percentage of your patient consultations is by appointment? | | About ______% | | | | |
|  | 33. Do you offer a walk-in hour? | | ❒ Yes ❒ No | | | | |
|  | 34. In the past 12 months, have you ever done the following to reduce financial obstacles to deprived patients:  1. Provide free samples of medication  2. Prescribe the cheapest equivalent medicine  3. Not charge the patient (e.g. for co- payments) | | ❒ Yes ❒ No  ❒ Yes ❒ No  ❒ Yes ❒ No | | | | |
|  | 35. In the past 12 months, how often have you noticed that patients delayed their visits for financial reasons? | | ❒ Frequently ❒ Occasionally ❒ Never | | | | |
|  | 36. If new patients enter your practice, do you receive their medical records from their previous doctor? | | ❒ Yes, always or usually  ❒ Only occasionally  ❒ Rarely or never | | | | |
|  | 37. Which restrictions do you apply on taking new patients? (More than one answer possible) | | ❒ No restrictions (everyone is accepted)  ❒ No new patients are taken above a maximum number  ❒ No new patients are taken above certain age  ❒ No new patients are taken outside geographical working area  ❒ I use a wait period for new patients  ❒ Acceptance depends on patients’ medical history  ❒ Acceptance depends on patients’ insurance status | | | | |
|  | 38. Do you provide health care to people, when you are not remunerated for this (for instance uninsured, illegal immigrants)? | | ❒ Yes, always  ❒ Only in urgent cases  ❒ No  ❒ No such people show up in my practice  ❒ Not applicable (in this country care for these people is remunerated) | | | | |
|  | 39. Do your medical files normally include the following information:  (Tick all that apply) | | ❒ Living situation  ❒ Ethnicity  ❒ Patients’ family history (e.g. depression, cancer)  ❒ Patients’ weight and height  ❒ Smoking  ❒ Blood pressure  ❒ Reason for encounter  ❒ Diagnosis  ❒ Prescribed medications  ❒ Test results | | | | |
|  | 40. How do you keep patient medical records? (Please tick only one answer) | | ❒ I keep records except for minor or trivial complaints  ❒ I keep records of all regularly attending patients  ❒ I keep records, unless it is too busy  ❒ I keep records routinely of all patient contacts  ❒ Don’t know | | | | |
|  | 41. In the past 2 years, have you used your medical record system to list a selection of patients on the basis of age, diagnosis or risk?  (Tick all that apply) | | ❒ Not applicable (I don’t use a computer for my medical records)  ❒ By age group (e.g. those above age 50)  ❒ By diagnosis or health risk (e.g. diabetes or hypertension)  ❒ By medications they take (e.g., patients on multiple medications)  ❒ Who need a reminder for prevention or follow-up | | | | |
|  | 42. For which of the following purposes do you use a computer in your practice?  (Tick all that apply) | | ❒ Not applicable (I don’t use a computer)  ❒ Making appointments  ❒ Issuing invoices  ❒ Issuing medicine prescriptions  ❒ Keeping records of consultations  ❒ Sending referral letters to medical specialists  ❒ Searching medical information on the internet  ❒ Sending prescriptions to the pharmacy | | | | |
|  | 43. How often do you meet face-to-face with the following professions (both professionally and socially):  1. Other GP  2. Practice nurse  3. Ambulatory medical specialist  4. Hospital medical specialist  5. Pharmacist  6. Home care nurse  7. Midwife  8. Physiotherapist  9. Social worker  10. Dietician | | Seldom Every 1-3 More than  or never months once a month  ❒ ❒ ❒  ❒ ❒ ❒  ❒ ❒ ❒  ❒ ❒ ❒  ❒ ❒ ❒  ❒ ❒ ❒  ❒ ❒ ❒  ❒ ❒ ❒  ❒ ❒ ❒ | | | | |
|  | 44. How often do you ask advice (e.g. by telephone) from the following medical specialists?  1. Paediatrician  2. Internist  3. Gynaecologist  4. Surgeon  5. Neurologist  6. Dermatologist  7. Geriatrician  8. Psychiatrist/ mental health professional  9. Radiologist | | Seldom Every 1-3 More than  or never months once a month    ❒ ❒ ❒  ❒ ❒ ❒  ❒ ❒ ❒  ❒ ❒ ❒  ❒ ❒ ❒  ❒ ❒ ❒  ❒ ❒ ❒  ❒ ❒ ❒  ❒ ❒ ❒    ❒ ❒ ❒ | | | | |
|  | 45. Does your practice nurse or assistant independently provide:  1. Immunisation  2. Health promotion (e.g. giving lifestyle or smoking cessation advice)  3. Routine checks of chronically ill patients (e.g. diabetes)  4. Minor procedures (e.g. ear syringing, wound treatment) | | ❒ Not applicable (No nurse in my practice)  ❒ Yes ❒ No  ❒ Yes ❒ No  ❒ Yes ❒ No  ❒ Yes ❒ No | | | | |
|  | 46. To what extent do you use referral letters (including details on provisional diagnosis and possible test results) when you refer patients to a medical specialist? I use letters: | | ❒ for all referred patients  ❒ for most referred patients  ❒ for a minority of referred patients  ❒ seldom or never | | | | |
|  | 47. To what extent do medical specialists inform you after they have finished the treatment or diagnostics of your patients? | | ❒ (Almost) always  ❒ Usually  ❒ Occasionally  ❒ Seldom or Never | | | | |
|  | 48. After a patient has been discharged, how long does it usually take to receive a (summary) discharge report from the hospital most frequented by your patients? | | ❒ 1-4 days  ❒ 5-14 days  ❒ 15-30 days  ❒ More than 30 days  ❒ I rarely or never receive a discharge report | | | | |
|  | 49. To what extent will patients in your practice population (people who normally apply to you for primary medical care) have you as the doctor of first contact for the following health problems? (This is only about the first contact, not about further diagnosis or treatment).    1. Child with severe cough  2. Child aged 8 with hearing problem  3. Woman aged 18 asking for oral contraception  4. Man aged 24 with stomach pain  5. Man aged 45 with chest pain  6. Woman aged 50 with a lump in her breast  7. Woman aged 60 with deteriorating vision  8. Woman aged 60 with polyuria  9. Woman aged 60 with acute symptoms of paralysis/paresis  10. Man aged 70 with joint pain  11. Woman aged 75 with moderate memory problems  12. Man aged 35 with sprained ankle  13. Man aged 28 with a first convulsion  14. Anxious man aged 45  15. Physically abused child aged 13  16. Couple with relationship problems  17. Woman aged 50 with psycho-social problems  18. Man aged 32 with sexual problems  19. Man aged 52 with alcohol addiction problems | | Almost Usually OccasionallySeldom/  always never  ❒ ❒ ❒ ❒  ❒ ❒ ❒ ❒  ❒ ❒ ❒ ❒  ❒ ❒ ❒ ❒  ❒ ❒ ❒ ❒  ❒ ❒ ❒ ❒  ❒ ❒ ❒ ❒  ❒ ❒ ❒ ❒  ❒ ❒ ❒ ❒  ❒ ❒ ❒ ❒  ❒ ❒ ❒ ❒  ❒ ❒ ❒ ❒  ❒ ❒ ❒ ❒  ❒ ❒ ❒ ❒  ❒ ❒ ❒ ❒  ❒ ❒ ❒ ❒  ❒ ❒ ❒ ❒  ❒ ❒ ❒ ❒  ❒ ❒ ❒ ❒  ❒ ❒ ❒ ❒ | | | | |
| 50. To what extent are you involved in the treatment and follow-up of patients in your practice population with the following diagnoses (“practice population” means: people who normally apply to you for primary medical care)?  1. Chronic bronchitis/ COPD  2. Hordeolum (Stye)  3. Peptic ulcer  4. Herniated disc lesion  5. Congestive heart failure  6. Pneumonia  7. Peritonsilar abscess  8. Parkinson’s disease  9. Uncomplicated diabetes (type II)  10. Rheumatoid arthritis  11. Depression  12. Myocardial infarction | | (Almost) Usually Occasionally Seldom/  always Never  ❒ ❒ ❒ ❒  ❒ ❒ ❒ ❒  ❒ ❒ ❒ ❒  ❒ ❒ ❒ ❒  ❒ ❒ ❒ ❒  ❒ ❒ ❒ ❒  ❒ ❒ ❒ ❒  ❒ ❒ ❒ ❒  ❒ ❒ ❒ ❒  ❒ ❒ ❒ ❒  ❒ ❒ ❒ ❒  ❒ ❒ ❒ ❒  ❒ ❒ ❒ ❒ | | | | |  |
| 51. To what extent are the following activities carried out in your practice population by you (or your staff) and not by a medical specialist? (Practice population means: people normally applying to you for primary medical care). For example, if wedge resections are (almost) always done by you, tick the appropriate box.  1. Wedge resection of ingrown toenail  2. Removal of sebaceous cyst from the hairy scalp  3. Wound suturing  4. Excision of warts  5. Insertion of IUD  6. Fundoscopy  7 Joint injection  8. Strapping an ankle  9. Cryotherapy (warts)  10.Setting up an intravenous infusion | | (Almost) Usually Occasionally Seldom/  always Never  ❒ ❒ ❒ ❒  ❒ ❒ ❒ ❒  ❒ ❒ ❒ ❒  ❒ ❒ ❒ ❒  ❒ ❒ ❒ ❒  ❒ ❒ ❒ ❒  ❒ ❒ ❒ ❒  ❒ ❒ ❒ ❒  ❒ ❒ ❒ ❒  ❒ ❒ ❒ ❒ | | | | |  |
| 52. When do you, or your staff, measure blood pressure?  (more than one answer possible) | | ❒ In connection with relevant clinical conditions  ❒ On request  ❒ Routinely in surgery contacts with adults (regardless of the reason for visit)  ❒ In adults when invited for this purpose | | | | |  |
| 53. When do you, or your staff, measure blood cholesterol level? (more than one answer possible) | | ❒ In connection with relevant clinical conditions  ❒ On request  ❒ Routinely in surgery contacts with adults (regardless of the reason for visit)  ❒ In adults when invited for this purpose  ❒ No such measures | | | | |  |
| 54. To what extent are you involved in health education as regards to the following topics: (More than one answer possible)  1. Smoking  2. Diet  3. Problematic use of alcohol  4. Physical exercise | | Not involved In connection with In group sessions or  normal patient contacts special programmes  ❒ ❒ ❒  ❒ ❒ ❒  ❒ ❒ ❒  ❒ ❒ ❒ | | | | |  |
| 55. Are you or your practice staff involved in the following activities?  1. Routine antenatal care  2. Immunisation of children (as part of a programme)  3. Paediatric surveillance of children  under 4 years  4. Influenza vaccination (as part of a programme)  5. Palliative care | | Involved Not involved  ❒ ❒  ❒ ❒  ❒ ❒  ❒ ❒  ❒ ❒ | | | | |  |
| 56. During the past 12 months, have you offered (a) special session(s) or clinics for the following groups?  1. Diabetic patients  2. Hypertensive patients  3. Pregnant women  4. Elderly | | Yes No  ❒ ❒  ❒ ❒  ❒ ❒  ❒ ❒ | | | | |  |
| 57. If you were confronted through your patient contacts with the following occurrences, would you report this (for instance to an authority)?  1. Repeated accidents in an industry  2. Frequent respiratory problems in patients living near a certain industry  3. Repeated cases of food poisoning among people living in a certain district | | Yes Probably Probably No Don’t know  Yes not  ❒ ❒ ❒ ❒ ❒  ❒ ❒ ❒ ❒ ❒  ❒ ❒ ❒ ❒ ❒ | | | | |  |
| 58. In the past 12 months, about how many weeks altogether have you been away from the practice due to:  1. Attending conferences or other educational activities  2.Research activities  3. Vacations  4. Illness | | ___ weeks  ___ weeks  ___ weeks  ___ weeks | | | | |  |
| 59. To what extent do you agree with the following statements?  1. I feel that some parts of my work do not really make sense  2. My work still interests me as much as it ever did  3. My work is overloaded with unnecessary administrative detail  4. I have too much stress in my current job  5. Being a GP is a well respected job  6. In my work there is a good correspondence between effort and reward | | Strongly Agree Disagree Strongly  Agree disagree  ❒ ❒ ❒ ❒  ❒ ❒ ❒ ❒  ❒ ❒ ❒ ❒  ❒ ❒ ❒ ❒  ❒ ❒ ❒ ❒  ❒ ❒ ❒ ❒  ❒ ❒ ❒ ❒ | | | | |  |

*Thank you for filling out this questionnaire!*
