# Supplementary material for: Process quality indicators in family medicine: results of an international comparison
Source: BMC Fam Pract. 2015 Dec 2;16:172. doi: 10.1186/s12875-015-0386-7 (PMC4667500; doi:10.1186/s12875-015-0386-7)
Supplement: Additional file 2: — (DOCX 19 kb) [file 12875_2015_386_MOESM2_ESM.docx]

| **Country** | **Ethics committee** |
| --- | --- |
| **Austria** | Ethics committee of the Medical University of Vienna |
| **Belgium** | University Hospital Ghent - Commission for Medical Ethics |
| **Bulgaria** | The coordinator sent an official letter to the Ministry of Health which gave consent and support for the survey. The coordinator confirmed that there is no statutory requirement for ethical approval for this study. |
| **Cyprus** | National Bioethical Committee of Cyprus |
| **Czech Republic** | General University Hospital linked to the First Faculty of Medicine, Charles University in Prague |
| **Denmark** | The coordinator confirmed that there is no statutory requirement for ethical approval for this study. |
| **Estonia** | The national coordinator consulted with the Ethics Review Committee on Human Research of the University of Tartu. It was confirmed that there is no statutory requirement for ethical approval for this study. |
| **Finland** | The ethical committee of Pirkanmaa Hospital District. |
| **Germany** | Ethics Commision of the "Landesärtzenkammer Hessen". |
| **Greece** | Bioethical committees of seventy hospital. |
| **Hungary** | National Ethical Committee |
| **Iceland** | The Icelandic Bioethics Committee. A national committee under the Ministry of Welfare. |
| **Ireland** | Irish College of General Practitioners Research Ethics Committee – National Committee. |
| **Italy** | At Local Health Authorities level. Approval was requested from LHA Ethical Committees. |
| **Latvia** | Latvian Physicians Association Board of Certification |
| **Lithuania** | Kauno Regionus Biomedicininu Tyrimu Etikos Komitetas |
| **Luxembourg** | National committee of Research Ethic (CNER) in Luxembourg |
| **Malta** | University of Malta Research Ethics Committee |
| **Netherlands** | The ethics committee of VU Medisch Centrum confirmed via an official letter that the research is outside the scope of the WMO Act (Medical Research Involving Human Subjects Act). |
| **Norway** | The coordinator confirmed that there is no statutory requirement for ethical approval for this study. |
| **Poland** | Bioethics approval of Jagiellonian University |
| **Portugal** | Ethical committee of Lisbon and Oporto regions.; the National Commission for Health Data Safety. |
| **Romania** | Scientific Committee of CPSS |
| **Spain** | Research Units of Primary Care of the Autonomous Community in the Basque Country. In all other Autonomous Communities the study was approved at the Healthcare Area level. |
| **Slovakia** | The national coordinator consulted with the Council of the Slovak Society of General Practice. It was confirmed that there is no statutory requirement for ethical approval for this study. |
| **Slovenia** | National medical ethics committee |
| **Sweden** | Regional Research Ethics Committte |
| **Switzerland** | Ethical Committee of the University of Lausanne |
| **Turkey** | Ethical committee of Kartal Research and Education Hospital in Istanbul |
| **United Kingdom** | University of Lincoln School of Health and Social Care Ethics Committee; National Research Ethics Service |
